# Comparing RMSEA-Based Indices for Assessing Measurement Invariance in Confirmatory Factor Models

**DOI:** 10.1177/00131644231202949

**Published:** 2023-11-01

**Authors:** Nataly Beribisky, Gregory R. Hancock

**Affiliations:** 1 York University, Toronto, Ontario, Canada; 2 University of Maryland, College Park, USA

**Keywords:** measurement invariance, confirmatory factor analysis, RMSEA, fit index

## Abstract

Fit indices are descriptive measures that can help evaluate how well a confirmatory factor analysis (CFA) model fits a researcher’s data. In multigroup models, before between-group comparisons are made, fit indices may be used to evaluate measurement invariance by assessing the degree to which multiple groups’ data are consistent with increasingly constrained nested models. One such fit index is an adaptation of the root mean square error of approximation (RMSEA) called RMSEA_D_. This index embeds the chi-square and degree-of-freedom differences into a modified RMSEA formula. The present study comprehensively compared RMSEA_D_ to ΔRMSEA, the difference between two RMSEA values associated with a comparison of nested models. The comparison consisted of both derivations as well as a population analysis using one-factor CFA models with features common to those found in practical research. The findings demonstrated that for the same model, RMSEA_D_ will always have increased sensitivity relative to ΔRMSEA with an increasing number of indicator variables. The study also indicated that RMSEA_D_ had increased ability to detect noninvariance relative to ΔRMSEA in one-factor models. For these reasons, when evaluating measurement invariance, RMSEA_D_ is recommended instead of ΔRMSEA.

When researchers are interested in assessing how observed variables are associated with hypothesized latent constructs, they may invoke a confirmatory factor analysis (CFA) model to specify the relations of interest. Before the magnitude of those associations can be investigated, however, it is necessary to evaluate how well the identified CFA model will fit the researcher’s data. In this vein, there have been numerous descriptive measures, known as *fit indices*, proposed to evaluate the correspondence between model and data. One of the most common is the root mean square error of approximation (RMSEA; Steiger & Lind, 1980), but there are numerous others, including, but not limited to, the comparative fit index (CFI; Bentler, 1990), the standardized root mean squared residual (SRMR; Bentler, 1995), and the Tucker–Lewis index (TLI; Bentler & Bonett, 1980; Tucker & Lewis, 1973). All of these indices evaluate model fit from complementary perspectives, incorporating aspects of parsimony and performance relative to competing models.

CFA models can also be extended to a multigroup (MG) framework, whereby a CFA model of similar, if not identical, structure can be fit simultaneously to data from participants from different populations. Such models may be analyzed for a variety of purposes, including testing differences in factor variances, covariances, and/or means. Before any between-group comparisons are made, however, it is important to determine the degree of *measurement invariance* across all groups. Indeed, doing so might even be the focus of the investigation, such as evaluating the extent to which an instrument’s items measure a given construct comparably across different populations. Whatever the ultimate purpose, measurement invariance is usually assessed using a multistep process where each subsequent step introduces more parameter (e.g., loading) equality constraints between groups (Millsap, 2012), and assessing changes in the overall model fit. Following Meredith (1993), the steps in this process typically include evaluating *configural invariance* (equivalent factor structure across groups), *weak/metric invariance* (equivalent loading magnitude across groups), and *strong/scalar invariance* (equivalent intercept magnitude across groups); less commonly in practice *strict/residual invariance* (equivalent residual variance across groups) may also be assessed. As such, measures of model fit specifically for MG models are required.

Fortunately, versions of fit indices in single-group CFA models exist in the multiple group context as well, where they can be used to evaluate the extent to which the multiple groups’ data are consistent with the increasingly restrictive models associated with typical steps in the measurement invariance assessment process. Perhaps most common are fit indices based upon RMSEA and CFI, and with a specific focus on MG model fit, the respective change-based indices ΔRMSEA and ΔCFI (Cheung & Rensvold, 2002). ΔRMSEA and ΔCFI are, not surprisingly, the differences between two RMSEA values and two CFI values, respectively, arising from the comparison of two nested models, as in the steps of the measurement invariance process. One critical concern of using ΔRMSEA and ΔCFI is that both indices can be insensitive to model misspecification, thus failing to detect noninvariance (Savalei et al., 2023). That is, if the two values being compared are similar, the difference between them could be excessively small and thus potentially mask misspecification introduced in a more restricted model.

An alternative fit index that can be used for evaluating measurement invariance is RMSEA_D_ (Browne & du Toit, 1992; Savalei et al., 2023). As described in detail below, instead of taking the difference between RMSEA values from two models being compared, RMSEA_D_ arises from an adaptation of a single RMSEA formula in which the chi-square and degree-of-freedom differences are embedded within a single index. RMSEA_D_ was initially known as RDR (root deterioration per restriction; Browne & du Toit, 1992), although Browne, one of the index’s original creators, later recommended against its use due to its high sensitivity (MacCallum et al., 2006). Accordingly, the utilization of RMSEA_D_ has been inconsistent in both the applied and methodological literatures (see Savalei et al., 2023). Recently, however, it has been reintroduced and even recommended in place of ΔRMSEA (Savalei et al., 2023) precisely because of its increased sensitivity (relative to ΔRMSEA), especially in assessing measurement invariance as illustrated using a series of real data invariance testing examples. Finally, while other analogs to RMSEA_D_ exist, such as a CFI_D_ as an alternative to ΔCFI, Savalei et al. (2023) noted that these lack the increased sensitivity to misspecification offered by RMSEA_D_. For this reason, the current work will focus solely on RMSEA_D_.

The reintroduction of RMSEA_D_ into the literature (see Savalei et al., 2023) has primarily included examples that compare the performance of RMSEA_D_ and ΔRMSEA in the assessment of measurement invariance in previously published articles. To build upon these empirical examples and assess the performance of RMSEA_D_ and its relation to the respective change-based index ΔRMSEA more systematically, the present study consists of both derivations and population analyses for one-factor CFA models; these serve as the basis for, and generalize to, multifactor CFA models. In the first part of the study, the derivations allow for the expression of one fit index in terms of the other for any potential MG one-factor CFA model, helping to understand the mechanics of performance differences between RMSEA_D_ and ΔRMSEA as a function of number of indicators, pattern of noninvariance, and groups’ relative sample size. In the second part, population analyses serve to make the derivations more concrete by illustrating specific examples of MG one-factor CFA models that researchers might encounter in practice.

## RMSEA for Single Groups and Multiple Groups

ΔRMSEA and RMSEA_D_ are both based upon the RMSEA fit index. Returning to the single-group context, the sample RMSEA estimates the degree of model misfit within a population (Steiger & Lind, 1980). When a model is not a perfect representation of the population, its corresponding reference distribution is the noncentral **χ**^2^ distribution (given that other data assumptions are met), where the noncentrality parameter λ captures the degree of noncentrality within the population. When an SEM model is estimated with maximum likelihood (ML), the population RMSEA, RMSEA_pop_, is

(1) $$\mathrm{RMSE}A_{\mathrm{pop}}\;=\;\sqrt{\frac{F_{\mathrm{ML}}}{\mathrm{df}}}$$

where *df* refers to the model degrees of freedom and *F*_ML_ is the value of the ML discrepancy function when fit to the population data. For covariance structure models specifically,

(2) $$F_{\mathrm{ML}}=\ln|\hat{\Sigma }|\;-\;\ln\left|S\right|+\;\mathrm{tr}\left(S\;{\hat{\Sigma }}^{-1}\right)-\;p,$$

where $\hat{\Sigma }$ denotes the model-implied variance/covariance matrix, S denotes the observed variance/covariance matrix, and *p* corresponds to the number of observed variables. *F*_ML_ relates directly to the noncentrality of the expected **χ**^2^ distribution for fitting a model based on sample size *N*, such that (*N*− 1)*F*_ML_*=*λ, and in turn *F*_ML_*=*λ/(*N*− 1). Thus, the greater the model misfit, the higher the noncentrality in the population, and hence the higher the value of RMSEA_pop_. For random samples from that population, the sample estimate for RMSEA in single-group models may be computed as

(3) $$\mathrm{RMSEA}=\sqrt{\frac{{\hat{F}}_{\mathrm{ML}}}{\mathrm{df}}}=\sqrt{\frac{\hat{\lambda }}{\mathrm{df}(N\;-\;1)}}=\sqrt{\frac{\;T\;-\;\mathrm{df}}{\mathrm{df}(N\;-\;1)}}$$

where ${\hat{F}}_{\mathrm{ML}}$ is the sample-based estimate of the ML discrepancy function, *T* is the model test statistic for the sample (following a **χ**^2^ distribution under standard assumptions), and *T-df* is an estimate $\hat{\lambda }$ of the noncentrality parameter λ ). When the degrees of freedom exceed the test statistic, RMSEA is set equal to zero.

Of direct relevance here, the formula for RMSEA can be extended to accommodate MG models. In the MG context, RMSEA_MG_ estimates the degree of model misfit across all groups being modeled. At the population level, for *G* groups the RMSEA_MG_ based on Steiger (1998) may be expressed as

(4) $$\mathrm{RMSE}A_{\mathrm{pop}-\mathrm{MG}}\;=\;\sqrt{G}\sqrt{\frac{\lambda }{\mathrm{df}(N\;-\;G)}},$$

where *N* corresponds to the total sample size (i.e., for two groups *N* = *n*_1_+*n*_2_). This formula may also be adapted to estimate a sample RMSEA_MG_ (i.e., for MG models) with

(5) $$\mathrm{RMSE}A_{\mathrm{MG}}=\sqrt{G}\sqrt{\frac{\hat{\lambda }}{\mathrm{df}(N\;-\;G)}}=\sqrt{G}\sqrt{\frac{\;T\;-\;\mathrm{df}}{\mathrm{df}(N\;-\;G)}}.$$

As will be described below, RMSEA_MG_ can be adapted to assess measurement invariance, the degree to which measurement properties of an instrument with respect to a latent trait are the same across different populations (Millsap, 2012).

## Measurement Invariance

The multiple-group models of interest in the current study are those used for assessing measurement invariance. As mentioned earlier, the multistep invariance testing process typically involves the introduction of additional constraints at each step of the procedure, with each new set of constraints reflecting a more restrictive degree of parameter equality across groups. Models associated with each stage are then able to be compared. To start, as outlined by Meredith (1993), *configural invariance* is assessed in which all groups must hold the same pattern of free and fixed/zero factor loadings. Unacceptable fit in this stage precludes further assessment, given the failure to support a common model configuration within which further invariance would be evaluated. Given adequate fit, the next step assesses *weak/metric invariance*, in which factor loadings must be the same across groups, followed by the *strong/scalar invariance* step that adds equivalent intercepts across groups. Finally, albeit less common in practice, to assess *strict/residual invariance*, residual variances for each indicator must also be equal across groups. Below we discuss ways in which these models are compared from step to step to assess the degree of invariance for a given factor model.

### χ^2^ Difference Test

The **χ**^2^ difference test (also known as the likelihood ratio test) computes the difference between two nested models’*T* statistics, Δ*T*. Under standard data assumptions, this difference itself follows a **χ**^2^ distribution, with degrees of freedom equal to the difference between the nested models’ degrees of freedom. The **χ**^2^ difference test assesses whether a more constrained model fits the data statistically significantly worse than a model without the additional constraints. For example, a model with weak/metric invariance may be compared with a model with configural invariance to test whether the constraints imposed by equating the corresponding factor loadings across groups will fit statistically significantly worse than a model with configural invariance only.

The problems of using the **χ**^2^ difference test for evaluating measurement invariance have been well noted in the literature (e.g., Cheung & Rensvold, 2002; Counsell et al., 2020; Kelloway, 1995), drawing from both the test’s logic and its sensitivity. First, as researchers proceed through the steps of testing for measurement invariance, they are looking for evidence of whether to select the more constrained model (i.e., selecting the model with more equality constraints over the model with fewer constraints). However, because the **χ**^2^ difference test detects whether the more constrained model is of statistically worse fit than the less constrained model, researchers often use a *failure* to reject the test’s null hypothesis to infer that the more constrained model is actually true in the population. The problem here is one of logic, as one can only state that the more constrained model did not show evidence of significantly degrading fit, but can never state that they have evidence that the more constrained model fits the data equally well. On other hand, when sample sizes are large, the problem is one of oversensitivity: even substantively trivial deviations from perfect fit can lead the **χ**^2^ difference test to be statistically significant. For these reasons, which mirror those when assessing single-group model fit, other indices are often sought to expand the evaluation of MG model fit.

## ΔRMSEA

An alternate approach for assessing measurement invariance involves the use of fit index comparisons such as ΔRMSEA. To compute ΔRMSEA, RMSEA is calculated using the sample formula defined above for each of the nested models (e.g., a model with configural invariance is compared with a model with metric invariance),

(6) $$\Delta \mathrm{RMSEA}=\mathrm{RMSE}A_{2}-\mathrm{RMSE}A_{1}$$

(7) $$\Delta \mathrm{RMSEA}=\;\sqrt{G}\sqrt{\frac{\;T_{2}\;-\;df_{2}}{df_{2}(N\;-\;G)}}\;-\;\sqrt{G}\sqrt{\frac{\;T_{1}\;-\;df_{1}}{df_{1}(N\;-\;G)}}$$

where RMSEA_1_, and its associated *T*_1_ and *df*_1_, corresponds to the less restricted model and RMSEA_2,_ and its associated *T*_2_ and *df*_2_, to the more constrained model. And just as there have been cutoffs proposed for evaluating whether an RMSEA value is evidence of good fit in a single-group SEM model (e.g., Hu & Bentler, 1999), so too have there been cutoffs proposed for ΔRMSEA in the context of measurement invariance. Chen (2007) presented recommendations for ΔRMSEA that depend on total sample size, pattern of noninvariance, and group sample size equality. Specifically, .010 was recommended for testing metric invariance with *N*≤ 300, with all factor loadings in one group established to be higher than in the other, and unequal group sample sizes. A cutoff of .015 was recommended for evaluating metric invariance with *N >* 300, with approximately half of the factor loadings established to be higher in one group and the other half higher in the other group, and equal sample sizes. Unfortunately, one of the problems with the use of ΔRMSEA is that a model with high initial degrees of freedom (i.e., reflected as a large denominator in the RMSEA formula) will mask misspecification when it is compared with a more constrained nested model (Savalei et al., 2023). Specifically, RMSEA values from the two models will be quite similar due to the dilution of misfit by a high number of *df*, causing ΔRMSEA to be overly small. Such large-*df* situations are indeed quite common, in particular, when a latent variable has relatively many indicators.

## RMSEA_D_

An alternative to ΔRMSEA, RMSEA_D_, was initially presented by Browne and du Toit (1992) who introduced it as the RDR. They suggested that it be obtained by adapting the sample RMSEA formula presented above by changing the **χ**^2^ statistic and the degrees of freedom, that is,

(8) $$\mathrm{RMSE}A_{D}=\sqrt{\frac{\Delta T\;-\;\Delta \mathrm{df}}{\Delta \mathrm{df}\;(N\;-\;1)}}$$

where Δ *df* is the difference in the degrees of freedom between the nested models and Δ *T* is the difference between the test statistics of the nested models. RMSEA_D_ has also been extended into a MG framework, where the sample-based RMSEA_D_ formula adapted by Dudgeon (2004) is

(9) $$\mathrm{RMSE}A_{D-\mathrm{MG}}=\sqrt{G}\sqrt{\frac{\Delta T\;-\;\Delta \mathrm{df}}{\Delta \mathrm{df}\;(N\;-\;G)}}.$$

Importantly, RMSEA_D_ (for single and multiple groups) can perform differently from ΔRMSEA in many contexts. For example, because RMSEA_D_ is not calculated as a difference between two (nested) models’ RMSEA values, RMSEA_D_ will not become excessively small if those models have similar RMSEA values. Furthermore, when models have large degrees of freedom, sensitivity to misspecification may be masked in ΔRMSEA, but will not have the same effect on RMSEA_D_. As noted by Savalei et al. (2023), because RMSEA_D_ is an adaptation of the RMSEA formula, it can be interpreted in RMSEA units. In contrast, because ΔRMSEA is simply a difference between two (nested) models’ RMSEA values, it does not share this interpretational advantage (indeed, it is for this reason that distinct cut-off values have been developed for ΔRMSEA and other change-based fit indices). This added feature of RMSEA_D_ also allows for the construction of confidence intervals around this fit index, for single-group and MG models.

## Relation Between ΔRMSEA and RMSEA_D_

To illustrate how these two important fit indices relate to one another, consider a scenario in which measurement invariance is being assessed for a one-factor CFA model across *G* = 2 populations. Assuming configural invariance holds, metric invariance must next be evaluated by comparing the fit of the configural (C) model to a metric (M) model in which the two populations are constrained to have the same factor loadings. For ΔRMSEA, the formula in this case would be

(10) $$\Delta \mathrm{RMSE}A_{\mathrm{pop},M-C}=\mathrm{RMSE}A_{\mathrm{pop},M}-\mathrm{RMSE}A_{\mathrm{pop},C}.$$

Substituting the original RMSEA_pop_ formulas, and recognizing that because we have assumed configural invariance the fit of a configural model in the populations is perfect (i.e., $\lambda_{C\;}$ = 0), we get

(11) $$\Delta \mathrm{RMSE}A_{\mathrm{pop},M-C}=\sqrt{2}\sqrt{\frac{\lambda_{M}}{df_{M}(N\;-2)}}-\;\sqrt{2}\sqrt{\frac{\lambda_{C}}{df_{C}(N\;-\;2)}}\;=\;\sqrt{2}\sqrt{\frac{\lambda_{M}}{df_{M}(N\;-\;2)}}.$$

Rearranging the ΔRMSEA_pop,M-C_ formula to solve for λ _M_,

(12) $$\lambda_{M}=\;\frac{{\left(\Delta \mathrm{RMSE}A_{\mathrm{pop},M-C}\right)}^{2}\;df_{M}(N\;-\;2)}{2}\;=\;\frac{{\left(\mathrm{RMSE}A_{\mathrm{pop},M}\right)}^{2}\;df_{M}(N\;-\;2)}{2}$$

Next, the fit index RMSEA_D_ for establishing metric invariance in the *G* = 2 population context can be defined as

(13) $$\mathrm{RMSE}A_{\mathrm{pop},D,M-C}=\sqrt{2}\sqrt{\frac{\Delta \lambda_{M-C}}{\Delta df_{M-C}(N\;-\;2)\;}},$$

where $\Delta \lambda_{M-C}$ and $\Delta df_{M-C}$ are the differences in the noncentrality parameters and degrees of freedom for population models assuming metric and configural invariance, respectively. However, because λ _C_ = 0, $\Delta \lambda_{M-C}$ reduces to λ _M_ and thus RMSEA_pop,D,M-C_ can be simplified to

(14) $$\mathrm{RMSE}A_{\mathrm{pop},D,M-C}=\sqrt{2}\sqrt{\frac{\lambda_{M}}{\Delta df_{M-C}(N\;-\;2)\;}}.$$

It now follows that RMSEA_pop,D,M-C_ can be expressed in terms of ΔRMSEA_pop,M-C_ by substituting the above expression for λ _M_:

(15) $$\mathrm{RMSE}A_{\mathrm{pop},D,M-C}=\sqrt{\frac{df_{M}\;(N\;-\;2)}{\Delta df_{M-C}(N\;-\;2)\;}}\;\Delta \mathrm{RMSE}A_{\mathrm{pop},M-C}=\;\sqrt{\frac{df_{M}}{\Delta df_{M-C}\;}}\;\Delta \mathrm{RMSE}A_{\mathrm{pop},M-C}$$

(16) $$\mathrm{RMSE}A_{\mathrm{pop},D,M-C}=\sqrt{\frac{df_{M}}{\Delta df_{M-C}\;}}\;\mathrm{RMSE}A_{\mathrm{pop},M}$$

The formula above demonstrates that ΔRMSEA_pop,M-C_ (which equals RMSEA_pop,M_) may be converted to RMSEA_pop,D,M-C_ simply through the square root of a ratio of degrees of freedom, assuming that the configural model is correct (and thus has a perfect fit in the population). Accordingly, the largest discrepancy between RMSEA_pop,D,M-C_ and ΔRMSEA_pop,M-C_ occurs when there is a large difference between $\Delta df_{M-C}$ and *df*_M_.

The difference between $df_{M}$ and $\Delta df_{M-C}$ can be magnified in numerous situations. For instance, in one-factor models with *p* indicators, $\Delta df_{M-C}$ is equal to *p*–1 (i.e., the number of constraints on non-scale-referent loadings). In contrast, as the reader can easily derive, $df_{M}$ is *p*^2^–2*p*–1. Thus, with an increasing number of indicators, *df*_M_ scales quadratically, while $\Delta df_{M-C}$ only scales linearly; as such, larger values of *p* result in larger expected values of RMSEA_D_ relative to ΔRMSEA_M-C_ for the same model. (Note that these principles can also extend to other invariance testing phases as well, such as the comparison between models with metric and scalar invariance.)

Although the derivation above can be useful in demonstrating the differences between ΔRMSEA and RMSEA_D_ generally, it may also be valuable to demonstrate actual differences between the two indices by employing examples similar to those found in practical research contexts. For this reason, we use the following population analyses to present several examples, showcasing how expected RMSEA_D_ and ΔRMSEA_M-C_ values are affected when different features of CFA models are manipulated (specifically, number of indicators, factor loading strength, and pattern of noninvariance). For the current focus on metric/weak invariance (i.e., not with intercepts as in scalar/strong invariance), population analysis allows for the specification of population covariance matrices along with group sample sizes, to evaluate some outcome of interest. In this case, we evaluated the values of ΔRMSEA_pop,M-C_ and RMSEA_pop,D.M-C_ across various patterns of noninvariance in two different populations. A population analysis is appropriate when sampling variability is not a focus in study (Bandalos & Leite, 2013), when the manipulation of distributions is not required, and/or when researchers are not directly interested in power and Type I error rates.

## Method

Table 1 presents all the conditions explored in the population analysis. For the one-factor model examined, the primary features manipulated were number of indicators (*p*), sample size ratio between groups, and pattern of measurement noninvariance. We chose to keep the context focused on two groups because, in practice, social science studies typically compare one reference group to one focal group (see Putnick & Bornstein, 2016). The one-factor models in our investigation had *p* = 4, 8, or 12 indicator variables; we started with *p* = 4 given that it is the minimum number required for a one-factor model to be over-identified (e.g., Bollen, 1989). The ratio of sample sizes in the population analyses was either 1:1 or 2:1. We incorporated a condition where sample size was unequal across groups, because, similar to the simulation study conducted by French and Finch (2006) who incorporated a condition of unequal sample sizes, we aimed to reflect the real-world condition in which less data are able to be collected for a focal group of interest than a reference group.

**Table 1:** Measurement Invariance Conditions for Population Analysis

*p*	IC	Group 1 standardized factor loadings	Group 2 standardized factor loadings
4	1	.5, .5, .5, .5	.5, .5, .5, .**4**
	1	.5, .5, .5, .5	.5, .5, .5, .**2**
	1	.5, .5, .5, .5	.5, .5, .5, .**6**
	1	.5, .5, .5, .5	.5, .5, .5, .**8**
	2	.5, .5, .5, .5	.5, .**4, .4, .4**
	2	.5, .5, .5, .5	.5, .**2, .2, .2**
	2	.5, .5, .5, .5	.5, .**6, .6, .6**
	2	.5, .5, .5, .5	.5, .**8, .8, .8**
	3	.5, .5, .5, .5	.5, .**2, .2, .8**
	3	.5, .5, .5, .5	.5, .**4, .4, .6**
	3	.5, .5, .5, .5	.5, .**2, .8, .8**
	3	.5, .5, .5, .5	.5, .**4, .6, .6**
8	1	.5, .5, .5, .5, .5, .5, .5, .5,	.5, .5, .5, .5, .5, .5, .5, .**4**
	1	.5, .5, .5, .5, .5, .5, .5, .5	.5, .5, .5, .5, .5, .5, .5, .**2**
	1	.5, .5, .5, .5, .5, .5, .5, .5	.5, .5, .5, .5, .5, .5, .5, .**6**
	1	.5, .5, .5, .5, .5, .5, .5, .5	.5, .5, .5, .5, .5, .5, .5, .**8**
	2	.5, .5, .5, .5, .5, .5, .5, .5	.5, .**4, .4, .4. .4, .4, .4, .4**
	2	.5, .5, .5, .5, .5, .5, .5, .5	.5, .**2, .2, .2, .2, .2, .2, .2**
	2	.5, .5, .5, .5, .5, .5, .5, .5	.5, .**6, .6, .6, .6, .6, .6, .6**
	2	.5, .5, .5, .5, .5, .5, .5, .5	.5, .**8, .8, .8, .8, .8, .8, .8**
	3	.5, .5, .5, .5, .5, .5, .5, .5	.5, .**2, .2, .2, .2, .8, .8, .8**
	3	.5, .5, .5, .5, .5, .5, .5, .5	.5, .**4, .4, .4, .4, .6, .6, .6**
	3	.5, .5, .5, .5, .5, .5, .5, .5	.5, .**2, .2, .2, .8, .8, .8, .8**
	3	.5, .5, .5, .5, .5, .5, .5, .5	.5, .**4, .4, .4, .6, .6, .6, .6**
12	1	.5, .5, .5, .5, .5, .5, .5, .5, .5, .5, .5, .5	.5, .5, .5, .5, .5, .5, .5, .5, .5, .5, .5, .**4**
	1	.5, .5, .5, .5, .5, .5, .5, .5, .5, .5, .5, .5	.5, .5, .5, .5, .5, .5, .5, .5, .5, .5, .5, .**2**
	1	.5, .5, .5, .5, .5, .5, .5, .5, .5, .5, .5, .5	.5, .5, .5, .5, .5, .5, .5, .5, .5, .5, .5, .**6**
	1	.5, .5, .5, .5, .5, .5, .5, .5, .5, .5, .5, .5	.5, .5, .5, .5, .5, .5, .5, .5, .5, .5, .5, .**8**
	2	.5, .5, .5, .5, .5, .5, .5, .5, .5, .5, .5, .5	.5, .**4, .4, .4, .4, .4, .4, .4, .4, .4, .4, .4**
	2	.5, .5, .5, .5, .5, .5, .5, .5, .5, .5, .5, .5	.5, .**2, .2, .2, .2, .2, .2, .2, .2, .2, .2, .2**
	2	.5, .5, .5, .5, .5, .5, .5, .5, .5, .5, .5, .5	.5, .**6, .6, .6, .6, .6, .6, .6, .6, .6, .6, .6**
	2	.5, .5, .5, .5, .5, .5, .5, .5, .5, .5, .5, .5	.5, .**8, .8, .8, .8, .8, .8, .8, .8, .8, .8, .8**
	3	.5, .5, .5, .5, .5, .5, .5, .5, .5, .5, .5, .5	.5, .**2, .2, .2, .2, .2, .2, .8, .8, .8, .8, .8**
	3	.5, .5, .5, .5, .5, .5, .5, .5, .5, .5, .5, .5	.5, .**4, .4, .4, .4, .4, .4, .6, .6, .6, .6, .6**
	3	.5, .5, .5, .5, .5, .5, .5, .5, .5, .5, .5, .5	.5, .**2, .2, .2, .2, .2, .8, .8, .8, .8, .8, .8**
	3	.5, .5, .5, .5, .5, .5, .5, .5, .5, .5, .5, .5	.5, .**4, .4, .4, .4, .4, .6, .6, .6, .6, .6, .6**

*Note. p* = number of observed variables; IC = measurement invariance condition where 1 = only the last loading is noninvariant; 2 = all but the first (scaling indicator) loading are noninvariant and homogeneous; 3 = all but the first (scaling indicator) loading are noninvariant and heterogeneous. Bold type denotes that the factor loading in group 2 is different from the factor loading in group 1.

Finally, we investigated three different patterns of noninvariance by manipulating the size of the loadings in the first group (the *focal* group), while keeping the loadings the same in the second group (the *reference* group) unchanged across all conditions. For each pattern of noninvariance examined, the first loading across both groups (with the factor and all variables in standardized metric) was always of identical magnitude (with a loading of 0.50) for scaling purposes. For the first pattern of noninvariance, all loadings except that of the last indicator (which had a loading of 0.20, 0.40, 0.60, or 0.80) were identical across focal and reference groups. For the second pattern of noninvariance, beyond the invariant scaling indicator, the magnitude of the remaining loadings in the focal group differed from the magnitude of the loadings in the reference group, with all nonscaling loadings in the focal group having the same magnitude (namely, 0.20, 0.40, 0.60, or 0.80). Finally, for the third pattern of noninvariance, the magnitude of the nonscaling loadings in the focal group also differed from the magnitude of those in the reference group, having one of two possible magnitudes (one higher than the loadings in the reference group, with loadings of 0.60 or 0.80 and one lower than the loadings in the reference group with loadings of 0.40 or 0.20). These three different patterns of noninvariance allowed us to use population analysis to determine both ΔRMSEA_pop,M-C_ and RMSEA_pop,D,M-C_ by imposing models with configural and metric invariance.

The population analysis was conducted using the lavaan package (Rosseel, 2012). When fitting all of the one-factor models, starting values were set at the true population parameters of the reference group.

## Results

**Figure 1:**
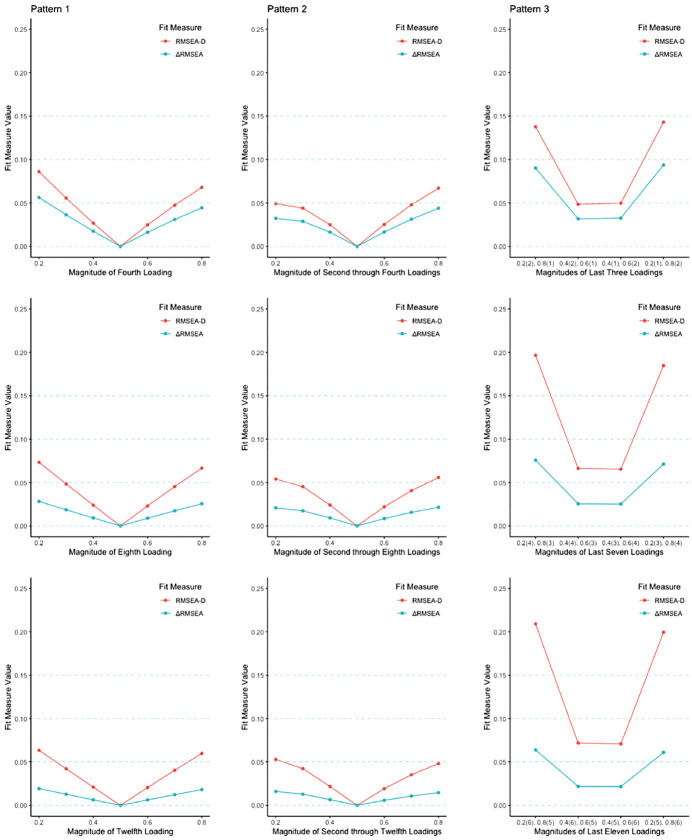
Population Analysis Results With Sample Size Ratio of 1:1 *Note.* Pattern 1 = only the last loading is noninvariant; Pattern 2 = all but the first (scaling indicator) loading are noninvariant and homogeneous; Pattern 3 = all but the first (scaling indicator) loading are noninvariant and heterogeneous. RMSEA = root mean square error of approximation.

In the sections below, results are first described by the three different patterns of noninvariance examined where group sample sizes are equal. The last section describes the association between ΔRMSEA_pop,M-C_ and RMSEA_pop,D,M-C_ and relative sample size, discussing the difference in the results when the sample size ratio is 1:1 versus 2:1. Accordingly, Figure 1 and Table 2 present the results for the population analyses when the sample size ratio is 1:1, that is, *n*_1_ = *n*_2_ (note that proxy sample sizes of *n*_1_ = *n*_2_ = 500 for the 1:1 scenario and *n*_1_ = 500 and *n*_2_ = 250 for the 2:1 scenario were chosen in order for the software to run; however, these values were arbitrary and irrelevant to the computation of all population RMSEA-based values).

**Table 2:** Population Analysis Results for 1:1 Sample Size Ratio

Pattern 1: Only the last loading is noninvariant				
*p*	RMSEA_pop,D,M-C_	ΔRMSEA_pop,M-C_	RMSEA_pop,D,M-C_ - ΔRMSEA_pop,M-C_	Last loading
4	.086	.056	.030	0.2
	.027	.018	.009	0.4
	.025	.016	.009	0.6
	.068	.044	.023	0.8
8	.073	.028	.045	0.2
	.024	.009	.015	0.4
	.023	.009	.014	0.6
	.067	.026	.041	0.8
12	.063	.019	.044	0.2
	.021	.006	.015	0.4
	.020	.006	.014	0.6
	.060	.018	.041	0.8
Pattern 2: All but the first (scaling indicator) loading are noninvariant and homogeneous				
*p*	RMSEA_pop,D,M-C_	ΔRMSEA_pop,M-C_	RMSEA_pop,D,M-C_ - ΔRMSEA_pop,M-C_	Loadings
4	.049	.032	.017	0.2
	.025	.016	.009	0.4
	.025	.017	.009	0.6
	.067	.044	.023	0.8
8	.054	.021	.033	0.2
	.024	.009	.015	0.4
	.022	.009	.014	0.6
	.056	.022	.034	0.8
12	.053	.016	.037	0.2
	.022	.007	.015	0.4
	.019	.006	.013	0.6
	.048	.015	.033	0.8
Pattern 3: All but the first (scaling indicator) loading are noninvariant and heterogeneous				
*p*	RMSEA_pop,D,M-C_	ΔRMSEA_pop,M-C_	RMSEA_pop,D,M-C_ - ΔRMSEA_pop,M-C_	Combination
4	.138	.090	.048	0.2(2), 0.8(1)
	.048	.032	.017	0.4(2), 0.6(1)
	.143	.094	.049	0.2(1), 0.8(2)
	.050	.033	.017	0.4(1), 0.6(2)
8	.197	.076	.121	0.2(4), 0.8(3)
	.066	.026	.041	0.4(4), 0.6(3)
	.185	.071	.113	0.2(3), 0.8(4)
	.065	.025	.040	0.4(3), 0.6(4)
12	.209	.064	.146	0.2(6), 0.8(5)
	.071	.022	.050	0.4(6), 0.6(5)
	.200	.061	.139	0.2(5), 0.8(6)
	.071	.021	.049	0.4(5), 0.6(6)

*Note.* RMSEA = root mean square error of approximation.

### Pattern 1: Only the Last Loading Is Noninvariant

For this pattern of noninvariance, the first *p*-1 loadings were identical between the reference and focal groups (i.e., 0.50), while the *p*th loading in the focal group was 0.20, 0.40, 0.60, or 0.80 (vs. the reference group loading of 0.50). In all instances, RMSEA_pop,D,M-C_ was larger than ΔRMSEA_pop,M-C_, with the difference being largest when the last loading of the focal group was most noninvariant (i.e., 0.20 or 0.80 as opposed to 0.40 or 0.60). With *p* = 4 indicators, for example, when the last loading in the focal group was 0.20 the difference between the indices was .030 (with ΔRMSEA_pop,M-C_ = .056 and RMSEA_pop,D,M-C_ = .086), whereas when the last focal group loading was 0.40, the difference between the two indices was .009 (with ΔRMSEA_pop,M-C_ = .018 and RMSEA_pop,D,M-C_ = .027).

As the number of indicators *p* increased, ΔRMSEA_pop,M-C_ was observed to decrease in magnitude. In contrast, RMSEA_pop,D,M-C_ would also decrease with an increasing number of indicators, but decreases with smaller in magnitude than those of ΔRMSEA_pop,M-C_. For instance, with a loading size of 0.80 and *p* = 4 indicators, ΔRMSEA_pop,M-C_ and RMSEA_pop,D,M-C_ were .044 and .068, respectively, whereas with *p* = 8, ΔRMSEA_pop,M-C_ dropped to .026 and RMSEA_pop,D,M-C_ held fairly steady at .067. Overall, this pattern of larger decreases in magnitude for ΔRMSEA_pop,M-C_ relative to RMSEA_pop,D,M-C_ led to the difference between the two indices being amplified with increasing number of indicators. This result is also illustrated in the first column of Figure 1. Moving down the column (i.e., increasing the number of indicators) highlights the different patterns between the two indices. Specifically, the line corresponding to RMSEA_pop,D,M-C_ maintains a V-shape with an increasing number of indicators. In contrast, the line corresponding to ΔRMSEA_pop,M-C_ has a V-shape that is flatter than the one associated with RMSEA_pop,D,M-C_ at *p* = 4, and becomes flatter still with an increasing number of variables. That is, as *p* increased ΔRMSEA_pop,M-C_ became less sensitive to detecting the single indicator’s noninvariance amid the larger number of invariant loadings, whereas RMSEA_pop,D,M-C_ tended to remain more sensitive, with this differentiation generally being most prominent when the last loading of the focal group was more noninvariant (0.20 or 0.80) rather than less so (0.40 or 0.60).

### Pattern 2: All But the First (Scaling Indicator) Loading Are Noninvariant and Homogeneous

In this pattern of noninvariance, beyond the invariant scaling indicator (which was again set to 0.50), the remaining loadings in the focal group differed identically from the loadings in the reference group, all being 0.20, 0.40, 0.60, or 0.80. When there was a greater difference in the loading size between the focal and reference groups’ 0.50 loading and the loadings of the focal group (0.20 or 0.80 rather than 0.40 or 0.60), values of both ΔRMSEA_pop,M-C_ and RMSEA_pop,D,M-C_ were generally larger, as expected. Furthermore, like in the first pattern of noninvariance, RMSEA_pop,D,M-C_ was always larger than ΔRMSEA_pop,M-C_, with the difference being more prominent when there was a greater difference in the size of the loadings between the reference and focal groups. For instance, with *p* = 4 indicators and a focal loading size of 0.20, ΔRMSEA_pop,M-C_ and RMSEA_pop,D,M-C_ were .032 and .049, respectively (with a difference of 0.17), whereas when the loading size was 0.40 ΔRMSEA_pop,M-C_ dropped to .016 and RMSEA_pop,D,M-C_ dropped to .025 (a difference of .009). Furthermore, similar to the first pattern, ΔRMSEA_pop,M-C_ decreased as the number of indicators increased, while RMSEA_pop,D,M-C_ would often, but not always, decrease with an increasing number of indicators, and those decreases were often smaller in magnitude than those of ΔRMSEA_pop,M-C_. This pattern can be viewed in the second column of Figure 1, where, like in the first pattern, the line associated with RMSEA_pop,D,M-C_ maintains a V-shape as the number of indicators increases. In contrast, ΔRMSEA_pop,M-C_ has a flatter V-shape than RMSEA_pop,D,M-C_ given *p =* 4, and continues to flatten with an increasing number of indicators. For example, when the focal group’s noninvariant loadings were 0.40, as *p* increased from 4 to 8 the ΔRMSEA_pop,M-C_ decreased from .016 to .009, while RMSEA_pop,D,M-C_ decreased from .025 to 024. Accordingly, with an increasing number of indicators, the difference between both indices was generally magnified.

### Pattern 3: All But the First (Scaling Indicator) Loading Are Noninvariant and Heterogeneous

For this pattern of noninvariance, like in Pattern 2, the value of the loadings in the focal group (other than the first loading) differed from the value of the loadings in the reference group. However, for this pattern, the nonscaling loadings had one of two possible magnitude combinations: (a) 0.20 and 0.80 or (b) 0.40 and 0.60. In all instances, the highest values for both ΔRMSEA_pop,M-C_ and RMSEA_pop,D,M-C_ resulted when the focal group’s loadings were a combination of 0.20 and 0.80 (rather than 0.40 and 0.60). This result is anticipated given that 0.20 and 0.80 are farther in magnitude from 0.50 (the loadings of the reference group) than 0.40 and 0.60.

Like the two other patterns of noninvariance examined, values of RMSEA_pop,D,M-C_ were always higher than values of ΔRMSEA_pop,M-C_. This difference was again more prominent when the loadings between the reference and focal groups were farther apart (here, in loading combinations of 0.20 and 0.80 rather than combinations of 0.40 and 0.60). For example, with *p* = 4, and two loadings set to 0.20 and one loading set to 0.80, the difference between ΔRMSEA_pop,M-C_ and RMSEA_pop,D,M-C_ was .048, with the values of the fit indices being .090 and .138, respectively. Changing the loading configuration such that two loadings were set to 0.40 and one loading was set to 0.60 (with *p* = 4) resulted in a .017 differences between the indices, where ΔRMSEA_pop,M-C_ and RMSEA_pop,D,M-C_ were equal to .032 and .048, respectively.

Once again, ΔRMSEA_pop,M-C_ decreased as the number of indicators increased. In contrast, RMSEA_pop,D,M-C_ increased with an increasing number of indicators, leading to the difference between both indices being greatly amplified with an increasing number of indicators. For example, with *p* = 4 and the combination of standardized factor loadings of two 0.20s and one 0.80, ΔRMSEA_pop,M-C_ and RMSEA_pop,D,M-C_ were equal to .090 and .138, respectively. When *p* increased to 8, and the combination of factor loadings was four loadings of 0.20 and three loadings of 0.80, ΔRMSEA_pop,M-C_ and RMSEA_pop,D,M-C_ were equal to .076 and .197, respectively. The difference between the fit indices was most obvious when the focal group had loading combinations of 0.20 and 0.80, as opposed to combinations of 0.40 and 0.60.

### Effect of Sample Size Ratio on Findings

Regardless of the specific sample size selected for a population analysis, when group sample sizes are equal (i.e., 1:1), RMSEA-based fit indices like those studied here will not change. This is because, although each group’s unique λ value can be obtained by multiplying the group’s associated discrepancy function by its sample size, and the total λ value associated with the MG model is obtained by summing the groups’ λ values, both ΔRMSEA_pop,M-C_ and RMSEA_pop,D,M-C_ require that the multisample λ be divided by the total sample size. Thus, in the aggregate represented by the MG fit indices, groups’ contributions are weighted not by sample size, but by sample size proportion. As such, all 1:1 sample size ratios will yield the same population fit, all 2:1 ratios will yield the same population fit (although typically different from 1:1 ratios), and so on. Table 3 illustrates the values of both fit indices when the sample size ratio was 2:1. Generally, the findings for the 2:1 sample size ratio had similar patterns to the results when groups were of equal size. In all conditions, RMSEA_pop,D,M-C_ was larger in magnitude than ΔRMSEA_pop,M-C_. For all patterns of invariance explored, the difference between the two fit indices was most evident when the loading differences between the reference and focal groups were greater (e.g., 0.20 and 0.80, rather than 0.40 and 0.60). Also, across all patterns of noninvariance, with an increasing number of indicators, values of ΔRMSEA_pop,M-C_ decreased; that is, it became less sensitive to detecting the target loading’s noninvariance in the context of increasing numbers of invariant loadings. Meanwhile, with an increasing number of indicators, values of RMSEA_pop,D,M-C_ consistently increased under the third pattern of noninvariance (all but the first loading are noninvariant and heterogeneous), and were largely decreasing across the other two patterns (with decreases tending to be smaller in magnitude than ΔRMSEA_pop,M-C_). That is, with increasing numbers of invariant indicators, RMSEA_pop,D,M-C_ did not illustrate a consistent pattern of decreased sensitivity as ΔRMSEA_pop,M-C_ so clearly did.

**Table 3:** Population Analysis Results for 2:1 Sample Size Ratio

Pattern 1: Only the last loading is noninvariant				
*p*	RMSEA_pop,D,M-C_	ΔRMSEA_pop,M-C_	RMSEA_pop,D,M-C_ - ΔRMSEA_pop,M-C_	Last loading
4	.079	.052	.027	0.2
4	.025	.016	.009	0.4
4	.024	.015	.008	0.6
4	.066	.043	.023	0.8
8	.069	.027	.042	0.2
8	.023	.009	.014	0.4
8	.022	.008	.013	0.6
8	.063	.024	.039	0.8
12	.060	.018	.041	0.2
12	.020	.006	.014	0.4
12	.019	.006	.013	0.6
12	.056	.017	.039	0.8
Pattern 2: All but the first (scaling indicator) loading are noninvariant and homogeneous				
*p*	RMSEA_pop,D,M-C_	ΔRMSEA_pop,M-C_	RMSEA_pop,D,M-C_ - ΔRMSEA_pop,M-C_	Loadings
4	.041	.027	.014	0.2
4	.023	.015	.008	0.4
4	.025	.016	.009	0.6
4	.070	.046	.024	0.8
8	.045	.017	.028	0.2
8	.022	.008	.013	0.4
8	.022	.008	.013	0.6
8	.058	.022	.036	0.8
12	.044	.013	.031	0.2
12	.020	.006	.014	0.4
12	.019	.006	.013	0.6
12	.050	.015	.034	0.8
Pattern 3: All but the first (scaling indicator) loading are noninvariant and heterogeneous				
*p*	RMSEA_pop,D,M-C_	ΔRMSEA_pop,M-C_	RMSEA_pop,D,M-C_ - ΔRMSEA_pop,M-C_	Combination
4	.122	.080	.042	0.2(2), 0.8(1)
4	.045	.029	.016	0.4(2), 0.6(1)
4	.144	.094	.050	0.2(1), 0.8(2)
4	.048	.031	.017	0.4(1), 0.6(2)
8	.186	.072	.114	0.2(4), 0.8(3)
8	.062	.024	.038	0.4(4), 0.6(3)
8	.184	.071	.113	0.2(3), 0.8(4)
8	.062	.024	.038	0.4(3), 0.6(4)
12	.201	.061	.140	0.2(6), 0.8(5)
12	.067	.020	.047	0.4(6), 0.6(5)
12	.197	.060	.137	0.2(5), 0.8(6)
12	.067	.020	.047	0.4(5), 0.6(6)

*Note.* RMSEA = root mean square error of approximation.

## Summary and Conclusion

There are various fit indices that exist to evaluate measurement invariance in MG models. One such example is ΔRMSEA, which takes the difference between RMSEA values corresponding to two nested models. ΔRMSEA has been critiqued for its lack of sensitivity which can occur, for example, when a model with high initial degrees of freedom is compared with a nested, more constrained model (Savalei et al., 2023). An alternative to ΔRMSEA is RMSEA_D_. RMSEA_D_ inserts the differences between the chi-squares and degrees of freedom associated with both nested models into an adapted RMSEA formula (Browne & du Toit, 1992; Savalei et al., 2023). RMSEA_D_ was recently reintroduced into the literature and recommended in place of ΔRMSEA, due to its increased sensitivity (Savalei et al., 2023).

To evaluate the performance of RMSEA_D_ and compare it with ΔRMSEA, the present study employed both derivations and a population analysis of one-factor models. The derivations illustrated how, given two nested models (one imposing configural invariance and one imposing metric invariance), an increasing number of indicators always leads to greater values of RMSEA_D_ relative to ΔRMSEA_M-C_ for the same model. The population analysis illustrated how RMSEA_pop,D,M-C_ had increased sensitivity relative to ΔRMSEA_pop,M-C_ in one-factor models with features similar to those found in practical research contexts. Specifically, values of RMSEA_pop,D,M-C_ were always greater than ΔRMSEA_pop,M-C_ values, the former more effective at detecting misspecification, especially when patterns of noninvariance were more extreme (greater differences between the loadings of the groups) and when there was a greater number of indicators. Accordingly, RMSEA_pop,D,M-C_ was more well-attuned to detecting greater misspecifications in noninvariance than ΔRMSEA_pop,M-C_.

To aid researchers in gauging whether a given value of ΔRMSEA is consistent with noninvariance, cutoffs such as .010 or .015 have been recommended for ΔRMSEA (the particular choice of cutoff depended on relative group size, sample size, and pattern of noninvariance, see Chen, 2007). As can be seen from the results of the population analysis, there were many instances where ΔRMSEA_pop,M-C_ fell below the .010 threshold. This occurred in tandem with RMSEA_pop,D,M-C_ being greater in magnitude, always more sensitive in detecting misspecification across the MG models.^1^

The increased sensitivity of RMSEA_D_ relative to ΔRMSEA can be explained by the relative dilution of misfit across degrees of freedom in the nested models being compared. For example, in a scenario where a model has a high number of initial degrees of freedom, the degrees of freedom associated with RMSEA_D_ will still be consistent with how many equality constraints are imposed by invoking a more restricted model. In contrast, ΔRMSEA will have misspecification diluted across a greater number of degrees of freedom, leading to its value being lower than that of RMSEA_D_.

Accordingly, both the derivations and the population analysis focused upon metric invariance, keeping equality constraints at the level of the factor loadings, without increasing constraints to the level of the intercepts. This was done, not because imposing scalar invariance is unimportant, but rather because the relative sensitivity of both indices can be illustrated without needing to evaluate measurement invariance at the level of the intercepts. In other words, it is possible to demonstrate the increased sensitivity of RMSEA_D_ relative to ΔRMSEA by simply focusing on assessing metric invariance, given that the relative dilution of misspecification across degrees of freedom will lead to greater values of RMSEA_D_ relative to ΔRMSEA regardless of whether loadings, intercepts, or residual variances are being evaluated for invariance. Thus, these findings should be directly applicable not only for scenarios where researchers are only interested in evaluating metric invariance, but in all steps of evaluating invariance, where these findings provide a foundation upon which the subsequent stages of invariance testing will only magnify the differences between both indices. Furthermore, although the population analysis only used one-factor models, the same principles can be extended to multifactor models, including longitudinal models, where the factors occur at different time points.

In sum, RMSEA_D_ was reintroduced into the literature due to its increased sensitivity to detect patterns of misspecification across MG models (see Savalei et al., 2023). This study employed both derivations and a population analysis to illustrate how the index is better at distinguishing various kinds of noninvariance than ΔRMSEA in different contexts. Due to its increased sensitivity, RMSEA_D_ is recommended over ΔRMSEA for evaluating measurement invariance.

## Notes

1. In both the derivation and the population analysis, we have assumed that the fit of the configural model was perfect ( $\lambda_{C}$ = 0). When the configural model is not of perfect fit, there are two scenarios that may result. First, $\lambda_{C}$ will be small and have little effect on the results. Second, $\lambda_{C}$ will be large and the assessment of measurement invariance would end at the configural model, making further evaluation is unnecessary.
